# Development of label-free electrochemical OMP-DNA probe biosensor as a highly sensitive system to detect of citrus huanglongbing

**DOI:** 10.1038/s41598-024-63112-w

**Published:** 2024-05-28

**Authors:** Hashem Kazemzadeh-Beneh, Mohammad Reza Safarnejad, Parviz Norouzi, Davood Samsampour, Seyed Mehdi Alavi, Davood Shaterreza

**Affiliations:** 1 Division of Biotechnology & Plant Molecular Genetic, Department of Horticulture Science, University of Hormozgan, Bandar Abbas, Iran; 2 Department of Plant Viruses, Agricultural Research Education and Extension Organization, Iranian Research Institute of Plant Protection, P.O. Box 1452-19395, Tehran, Iran; 3 Faculty of Chemistry, Center of Excellence in Electrochemistry, University of Tehran, Tehran, Iran; 4 National Institute for Genetic Engineering and Biotechnology, Tehran, Iran

**Keywords:** Huanglongbing, Citrus greening, OMP gene, DNA probe, Label free electrochemical biosensor, DNA hybridization, Biochemistry, Biological techniques, Biotechnology, Chemical biology, Molecular biology, Plant sciences

## Abstract

The fabrication of the first label-free electrochemical DNA probe biosensor for highly sensitive detection of *Candidatus Liberibacter asiaticus* (CLas), as the causal agent of citrus huanglongbing disease, is conducted here. An OMP probe was designed based on the hybridization with its target-specific sequence in the outer membrane protein (OMP) gene of CLas. The characterization of the steps of biosensor fabrication and hybridization process between the immobilized OMP-DNA probe and the target ssDNA oligonucleotides (OMP-complementary and three mismatches OMP or OMP-mutation) was monitored using cyclic voltammetry and electrochemical impedance spectroscopy based on increasing or decreasing in the electron transfer in [Fe (CN)_6_]^3−/4−^ on the modified gold electrode surface. The biosensor sensitivity indicated that the peak currents were linear over ranges from 20 to 100 nM for OMP-complementary with the detection limit of 0.026 nM (S/N = 3). The absence of any cross-interference with other biological DNA sequences confirmed a high selectivity of fabricated biosensor. Likewise, it showed good specificity in discriminating the mutation oligonucleotides from complementary target DNAs. The functional performance of optimized biosensor was achieved via the hybridization of OMP-DNA probe with extracted DNA from citrus plant infected with CLas. Therefore, fabricated biosensor indicates promise for sensitivity and early detection of citrus huanglongbing disease.

## Introduction

Over the past two decades, the industry of worldwide citrus production has been seriously threatened by citrus greening or huanglongbing (HLB) disease. HLB is a Gram-negative bacterium belonging to the α-Proteobacteria family, non-culturable, and phloem-restricted, which is associated with the three species of *Candidatus* Liberibacter (CL) genus including CL. Asiaticus (CLas), CL. americanus, and CL. Africanus^1^. Among them, the most widespread and economic losses in worldwide citrus production belong to the CL. asiaticus, which is vectored by the psyllid *Diaphorina citri* (Kuwayama) to new citrus-growing regions. Citrus trees infected by this pathogen become unproductive and, as time elapses, they may eventually death^2^. To save a billion-dollar industry from significant financial inferences from HLB infectious, the development of rapid detection, cost-effective, and highly sensitive techniques for specific identification of the HLB and subsequent management strategies to mitigate the disease have been prioritized as the main goals in the citrus industry and the regulatory agencies in citrus producing countries^3^.

Microscopy, serology, and DNA-DNA hybridization are the pioneer diagnostic techniques that have been used for HLB detection^4^. Also, the application of imaging techniques and extended spectral angle mapping (ESAM) for the detection and translocation of HLB disease has previously been established^5^. However, these techniques are time-consuming, labor-intensive, and lack proper sensitivity. Besides these, for the detection of HLB, polymerase chain reaction (PCR)-based molecular diagnostic techniques and protocols such as conventional PCR^6^, multiplex PCR^7^, quantitative real-time PCR^8^, nested competitive PCR^9,10^, droplet digital PCR^11^, immune capture-PCR^12^, Loop-mediated isothermal amplification (LAMP)^13^, and one-tube dual primer TaqMan® PCR^4^ have been employed. Requiring an accurate primer design, attempting to generate a comprehensive optimization of the test for amplifying conditions, having a high risk of contamination, decreasing sensitivity due to the interference of various factors, and relative decrease in sensitivity are some limitations to using PCR-based diagnostic techniques^14^. Moreover, they are time-consuming, costly, require advanced equipment and professional technicians. One the other hand, since all the above PCR assays have been used to detect in psyllid vectors, utilization of the mentioned-techniques for early detection of HLB is erratic due to the irregular distribution of HLB bacterial in symptomatic and non-symptomatic field-grown trees, seasonal variation of their titers, and the presence low titers of the bacterium in citrus plant tissues^15^. There is still a critical need to progress the selectivity, sensitivity and rapidity of HLB diagnosis.

Recently, several polyclonal^16^, monoclonal antibodies^17^and serological or/immunoassay tests based mentioned-antibody such as direct tissue blot immunoassay (DTBIA) and enzyme-linked immunosorbent assay (ELISA) have been established for HLB diagnosis. Although these antibodies have the advantage of high sensitivity, but they are time-consuming, laborious, expensive, and multistep process^18^. To boost pathogens detection systems, molecular techniques have been complicated in forming new tests that are trustworthy, affordable, and low-cost. Therefore, considering the sensitive and specific alternative methods to HLB detection is a vital challenge to progress choice-making in citrus greening control. Accordingly, electrochemical biosensors due to their high sensitivity, simplicity, high measuring speed, easy to operation, portability, and economical with potential for applications in sub-atomic sensing instruments, accuracy, and low cost can be considered as an alternative technique in contrast with previously applied techniques for HLB detection.

Currently, electrochemical biosensors are considered promising analytical platforms in a variety of applications such as human disease detection, environmental control, horticulture and plant biology, food safety, and forensic analysis^19^. Until now, several electrochemical biosensors for detecting plant pathogenic diseases including *Citrus tristeza virus*^20^, *Candidatus Phytoplasma Aurantifolia* as agents of witches’ broom disease^21^, Citrus bacterial canker disease^22^ and *Fig mosaic virus*^23^ have been reported. To-data, the gas biosensor arrays based on single-stranded DNA-functionalized single-walled carbon nanotubes for the detection of volatile organic compound biomarkers released HLB-infected citrus plants, microsensor (an organic electrochemical transistor functionalized with ceria nanoparticles) for limonin detection as an indicator of HLB disease and chemiresistive biosensor using single-walled carbon nanotubes for diagnosing a secreted protein biomarker of CLas have developed^24–26^. The mentioned diagnosis systems are indirect way based on profiling of plant-specific biochemical markers including gentisoyl glucoside, limonin, and volatile organic compound biomarkers (ethylhexanol, linalool, tetradecene, and phenylacetaldehyde), which are synthesized by HLB-infected citrus trees^27^. The sensitivity and accuracy of these systems can be affected by various factors such as temperature, environmental and soil conditions, type of citrus variety or cultivar, seasonal variation of HLB bacterial population, and their non-uniform distribution due to alternating the content of the measured biochemical marker, which leads to a decrease in their accuracy. Thus, the proper results of the diagnosis technique in HLB detection for one cultivar, may not be suitable for another, which is due to differences in their genetic background in responding against HLB biotic stress. These techniques will lose their analytical performance if these biochemical markers are synthesized in response to nutritional deficiencies and viral infection or even if they are not synthesized in some citrus cultivars. Therefore, the generation biosensor technique based on bacterial cells or DNA for the direct way detection of HLB can be an alternative approach to enhance the analytical performance of the diagnosis system.

In recent years, the placement of nucleic acid (dsDNA: double strand DNA or ssDNA: single strand DNA) as a recognition layer in DNA biosensors design has not only created a new area in clinical analysis but also their application in pharmacogenomics, drug screening, medical diagnosis, molecular diagnostics, food analysis, and environmental monitoring has greatly improved due to rapid and low-cost detection of specific DNA sequences^28,29^. DNA probes are short oligonucleotides (from 25 to 60 bp) which can hybridize with specific-target sequences. The DNA hybridization or hydrogen bonding event among a target specific complementary DNA sequence and a ssDNA probes layer immobilized on a transducer or working electrode surface followed by the formation of a stable DNA double helix complex are main principles for the design of electrochemical DNA biosensor^30^. Meanwhile, a good hybridization relies on applying the appropriate DNA probe immobilization techniques for improving a good orientation of the immobilized DNA probe to hybridize with its complementary DNA target on the working electrode surface^31^. However, the immobilization of DNA probes on the surface of electrodes is a vital step in the construction of electrochemical DNA biosensors with high sensitivity, specificity and, lifetime properties. Generally, three DNA probes immobilization strategies including the adsorption method, covalent bonding, and avidin–biotin interaction have been conducted successfully in electrochemical DNA biosensor^30^. Among them, the adsorption is the simplest method and the electrostatic adsorption between a negatively charged phosphate group of DNAs on the positively charged films-modified electrodes is the main factor for DNA probe immobilization on the electrode surface without the prerequisite for any chemical reagents. In the covalent bonding method, at the first step, the synthesized DNA probe is linked with the group of thiols (S–H) or amines (NH_2_) at the end of 3′ or 5′ oligonucleotides sequences, and in the next step through and covalent attachments covalently bonded to the metal surface of the electrode. Formation complex of avidin (either streptavidin)-biotin is key principle for non-covalent immobilization of DNA probes on the electrode surface in avidin/streptavidin–biotin interaction method^29,30^. Forming a self-assembled monolayer (SAM) of thiols is frequently used for covalently immobilization of DNA probes on the between thiol-modified DNA probes and gold (Au) surface^32^.

Development of electrochemical DNA biosensors according to DNA hybridization detection can be categorized into label-free and label-based methods. For label-free electrochemical DNA biosensors, the electrochemical signals resulting from the electroactive DNA bases underwent a reduction and oxidation current during or after hybridization with its target complementary DNA are detected. For indirect or label-based electrochemical DNA biosensors, DNA hybridization is detected by redox active molecules as indicators to improve electron transfer among the electroactive base and the electrode surface^30^. The outer membrane protein (OMP) is an outer membrane protein gene of CLas with highly conserved genomic loci that have been used as molecular markers in the detection of HLB^33,34^. In the present study, the development of the first label-free electrochemical OMP-DNA probe biosensor or genosensor for direct and highly sensitive detection of the bacteria agent of citrus greening is designated.

## Results

### Surface morphological characterization of the modified electrode

**Figure 1:**
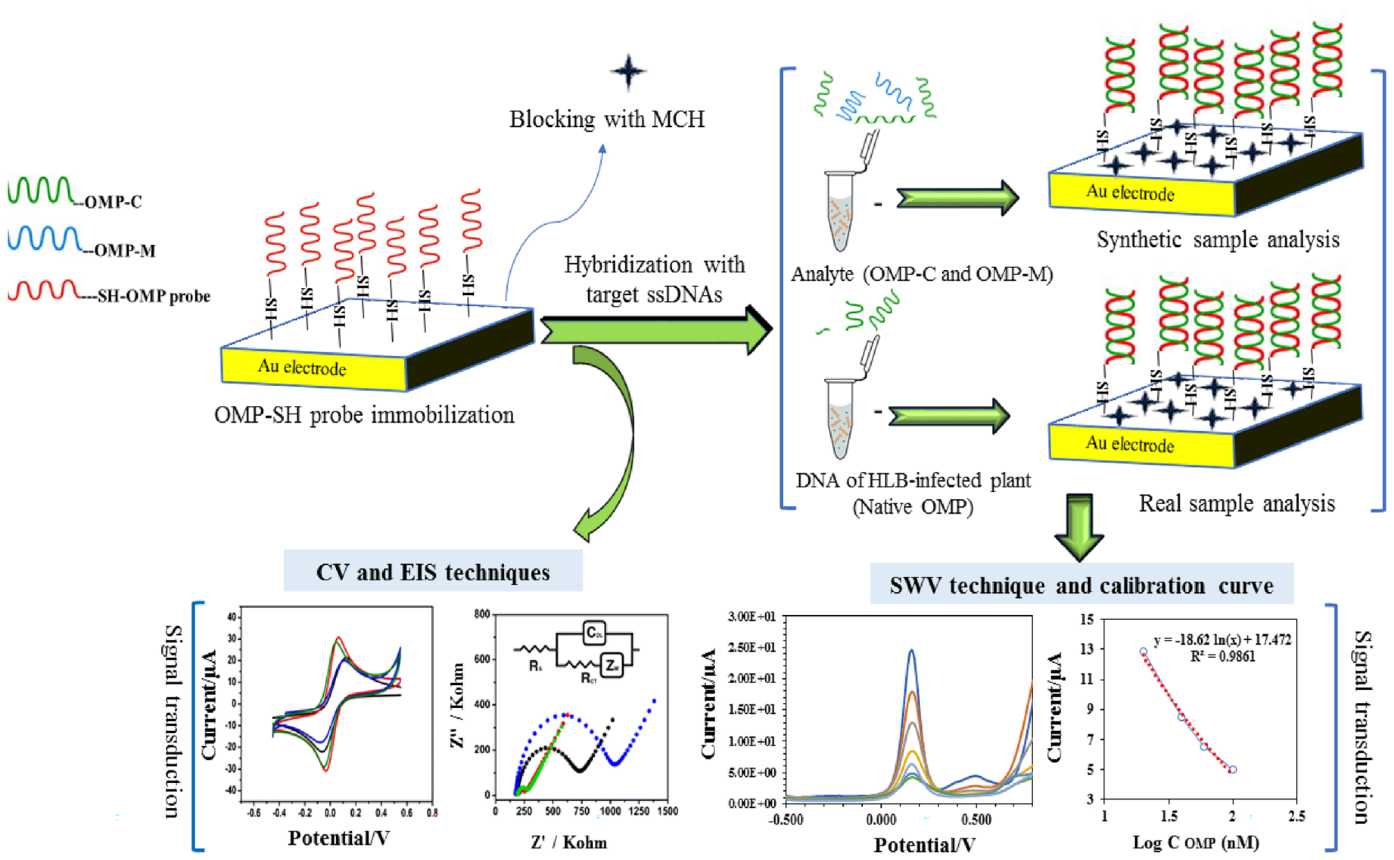
Schematic diagrams of the electrochemical OMP-DNA probe biosensor fabrication steps and performance. MCH: 6-Mercapto-l-hexanol; OMP-SH: outer membrane protein thiolated probe; OMP-M: OMP mutation consisted three-base mismatched DNA or three points mutation of OMP sequence; OMP-C: OMP-Complement; CV: cyclic voltammetry; SWV: square wave voltammetry; EIS: electrochemical impedance spectroscopy.

**Figure 2:**
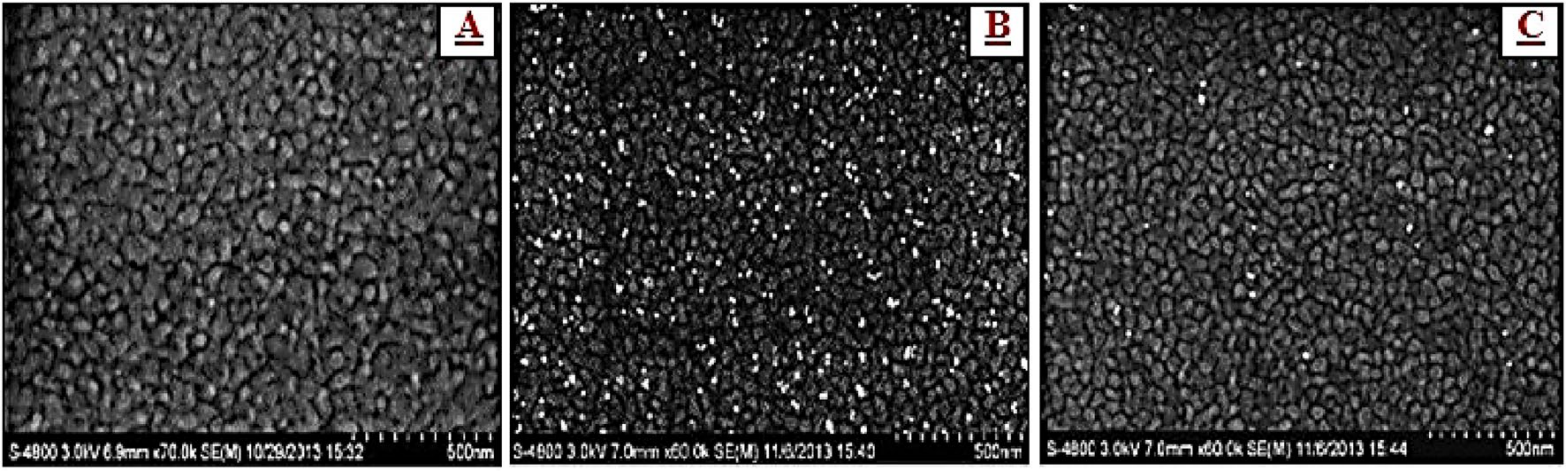
Surface morphological characterization of modified electrodes with OMP-SH probe/Au (**A**), OMP-SH probe/MCH/Au (**B**) and OMP-SH probe/MCH/OMP-C/Au (**C**) by using SEM images analysis.

Figure 1 displays the scanning electron microscope (SEM) images of OMP-SH probe/Au, OMP-SH probe/MCH/Au, and OMP-SH probe/MCH/OMP-C/Au modified electrodes. The surface morphology of the Au electrode modified with the OMP-SH probe exhibited the uniform distribution of sporadically grafted OMP-SH probe on the surface of the electrode and adequately covered the surface with ssDNA probe linked to thiol group (Fig. 2A). After immobilization process for the OMP-SH probe, it could be obviously seen that incubating with MCH blocked the un-assembled sites and restricted the non-specific adsorption on OMP-SH probe/MCH/Au surface, as observes to bright spots forms in Fig. 2B. The roughness and thickness surface of the OMP-SH probe/MCH/Au surface was fittingly enlarged by the hybridization of OMP-SH probe immobilized with the complementary OMP-C on the electrode surface (Fig. 2C). Actually, the formation of ssDNA probe-OMP-C DNA complex structure reached a homogeneous surface with a few bright spots on modified OMP-SH probe/MCH/OMP-C/Au electrode, confirming the successful hybridization event.

### Electrochemical characterization of OMP-SH probe/MCH/Au electrode

**Figure 3:**
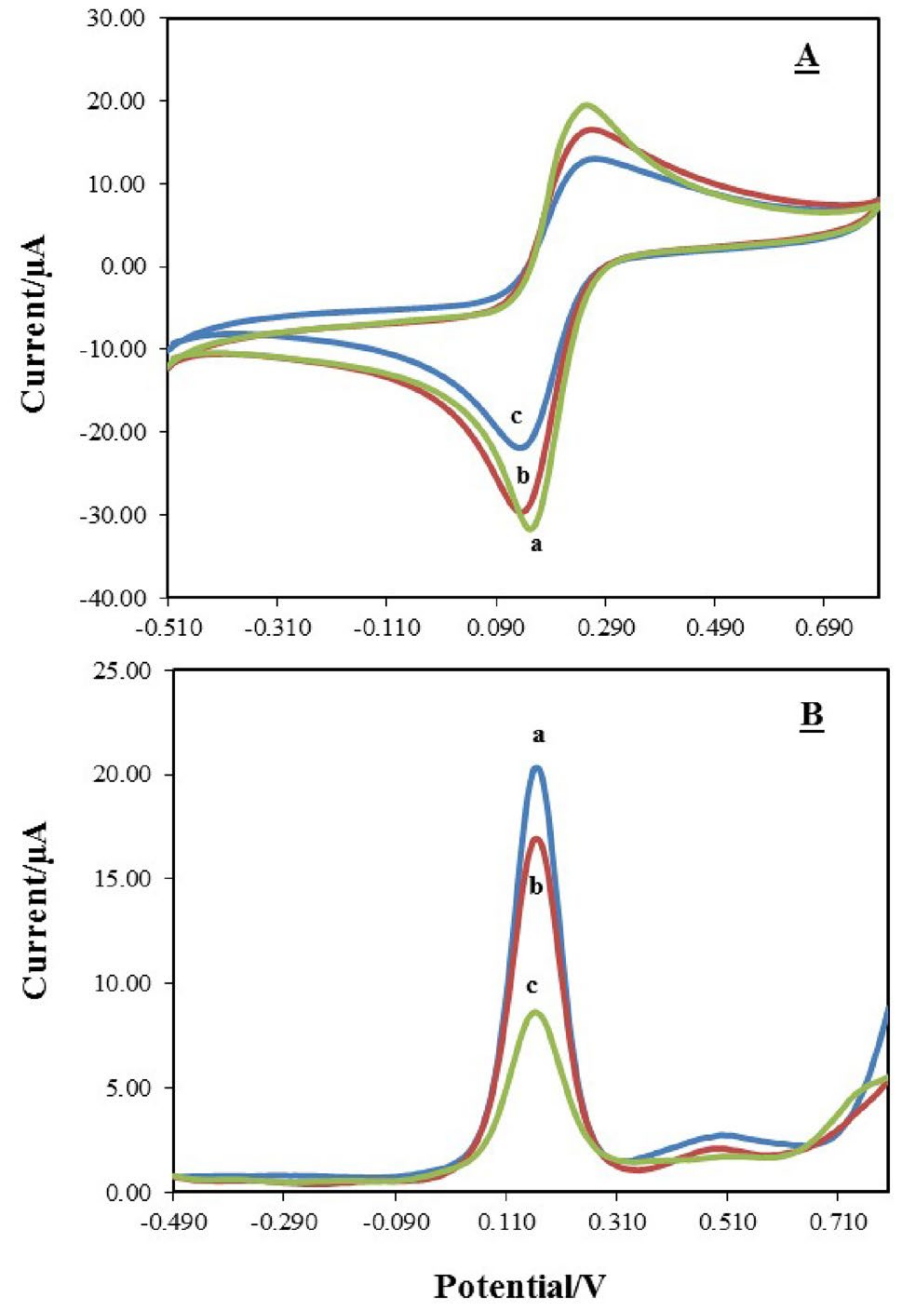
(**A**) Cyclic voltammogram resulting from electrochemical characterization of the OMP-SH probe/MCH/Au electrode by cyclic voltammetry and (**B**) square wave voltammograms resulting from before and after hybridization of OMP-C oligonucleotide onto OMP-SH probe/MCH/Au electrode by square wave voltammetry in PBS (phosphate-buffered saline) solution, pH 7.5, containing 250 mM KCl and 5 mM [Fe (CN)_6_]^3−/4−^. (**A**) bare Au (curve a), OMP-SH probe/Au (curve b) and OMP-SH probe/MCH/Au (curve c). (**B**) bare Au (curve a), OMP-SH probe/MCH/Au (curve b) and OMP-SH probe/MCH/OMP-C/Au (curve c).

Surface modification at each step of electrode fabrication was electrochemically inspected by cyclic voltammetry. The electrochemical behavior of the ferri/ferrocyanide redox couple due to electron transfer kinetics changes and reversibility at different electrodes has made it frequently used to explore the changes in electrode response after each surface modification step^35^. However, the CV was conducted in PBS solution containing 5 mM [Fe (CN)_6_]^3−/4−^ and 250 mM KCl with a pH 7.5 scan rate of 100 mV. Figure 3A indicates the cyclic voltammogram of the sequential modification of the bare Au (curve a), OMP-SH probe/Au (curve b), and OMP-SH probe/MCH/Au (curve c) electrodes. A pair of well-defined reversible peak currents were produced for the bare Au electrode, which indicates the high rate of electron transfer without any intervention at the expanded surface area of the electrode (curve a). After electrodeposition and immobilization of the OMP-SH probe and formation of a SAM, the peak currents and electron conductivity were remarkably lessened for the OMP-SH probe/Au electrode (curve b). This lessening in voltammogram could be associated with the blocking effect of the formed SAMs by the thiol group and limiting the diffusion [Fe (CN)_6_]^3−/4−^ redox couple toward the electrode surface due to the lower surface concentration for them, which signifies SAM formation. Likewise, when the MCH was incubated on the modified surface of the OMP-SH probe/Au electrode (curve c) to restrict the non-specific adsorption, the OMP-SH probe/MCH/Au electrode revealed a dramatic reduction of the peak current, which indicated the appreciative immobilization of OMP-SH probe. Therefore, the OMP-SH probe/MCH/Au electrode displayed less faradic currents compared to the OMP-SH/Au electrode.

### Electrochemical response of target DNAs and specific hybridization

The specificity of the OMP-DNA probe biosensor was analyzed by SWV for the detection of complementary ssDNA. Figure 3B displays the SWV voltammogram of the OMP-SH probe/MCH/Au electrode in the presence redox couple before and after incubation with 20 µl of 80 nM OMP-C oligonucleotide. The voltammogram obtained for the bare Au electrode showed a well-defined curve with much larger peak currents, indicating that it has no responses (curve a). The voltammogram for the OMP-SH probe/MCH/Au electrode exhibited a remarkable decrease in peak currents (curve b), followed by a further decrease or smaller peak current that appeared only after the dropping of OMP-C onto the electrode surface (curve c). This approves that the immobilized DNA probe had an obvious response for OMP-C target DNA via hybridization. During the hybridization of the OMP-SH probe immobilized with the complementary OMP-C on the electrode surface, a higher demand for electrons for the restructuration of the double-helix complex (dsDNA molecule) leads to an increase in the reduction potential through a modification in the current potential at the electrode surface with less electron availability for the molecule^36^. Thus, the hybridization event performs as an inert electron transfer blocking layer and an insulator layer against the diffusion of redox couple toward the electrode surface.

**Figure 4:**
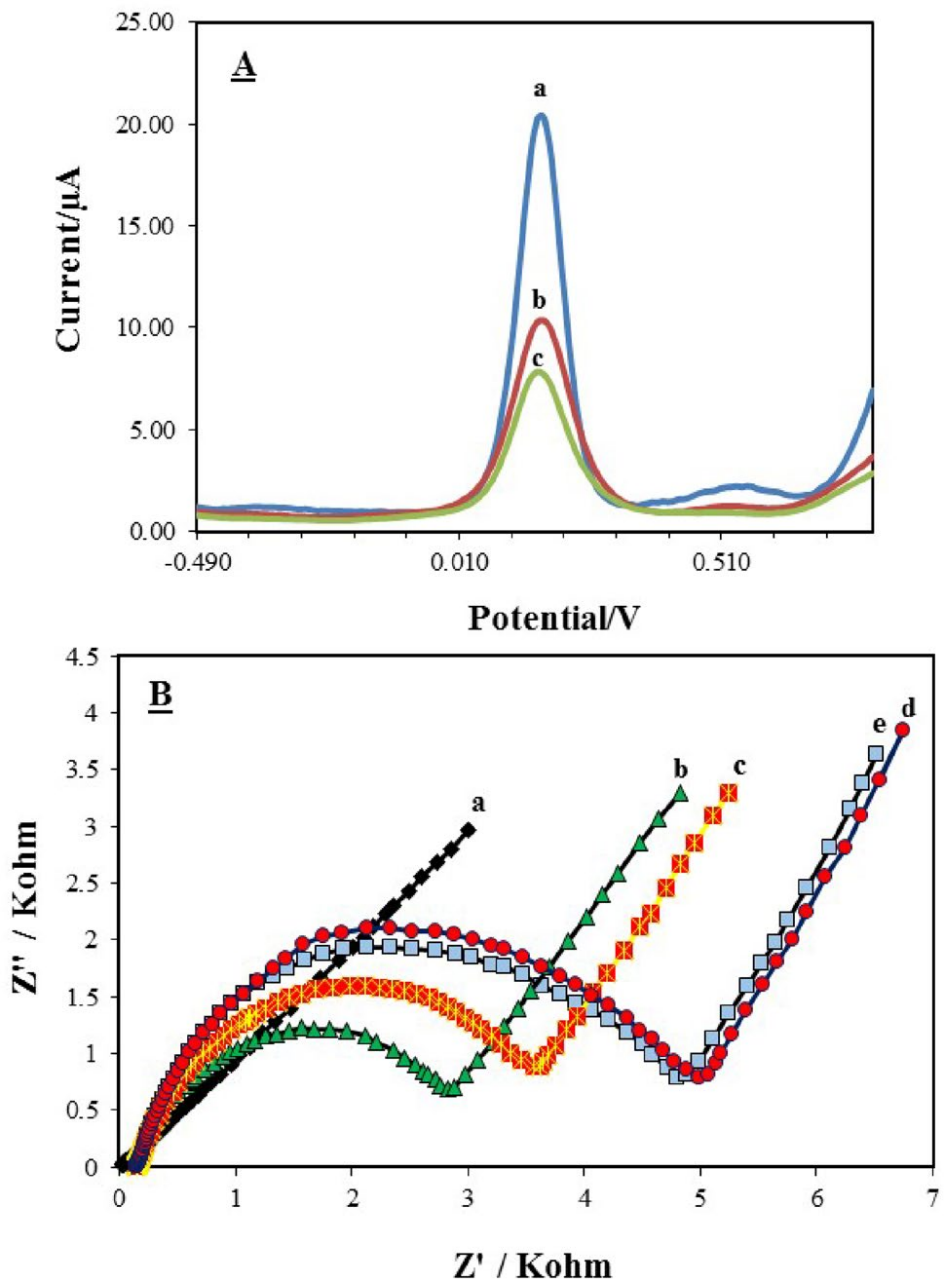
(**A**) Cross-interference assays of the fabricated OMP-DNA probe biosensor for the OMP-M (with base mutation or base mismatched DNA oligonucleotide at 80 nM concentration) and OMP-C (80 nM) in by square wave voltammetry and (**B**) electrochemical characterization of the different modified electrodes by electrochemical impedance spectroscopy in PBS (phosphate-buffered saline) solution, pH 7.5, containing 250 mM KCl and 5 mM [Fe (CN)_6_]^3−/4−^. (**A**) bare Au (curve a), OMP-SH probe/MCH/OMP-M/Au (curve b) and OMP-SH probe/MCH/OMP-C/Au (curve c). (**B**) bare Au (curve a), 1µM OMP-SH/Au (curve b), OMP-SH/MCH/Au (curve c), OMP-SH/MCH/OMP-C/Au (curve d) and OMP-SH/MCH/OMP-M/Au (curve e).

As can be seen in Fig. 4A, when the OMP-M oligonucleotide was cast on the modified OMP-SH probe/MCH/Au electrode, the resulting voltammogram indicated a peak current slightly larger than the peak current of OMP-C (curve c). Indeed, this slight increase in peak current is admirable evidence of the incomplete hybridization at base mismatched sites, which diminishes the generated-hybridization mass transfer barriers against the electron transfer toward the electrode surface. This shows that the fabricated biosensor has specificity and distinct response for the base mismatched OMP-M sequence compared to the OMP-C probe. The ΔI/I_0_ were 16.51% (RSD = 3.45%), 35.62% (RSD = 3.2%) and 49.57% (RSD = 2.82%) for OMP-SH probe, OMP-M and OMP-C, respectively. This approves the high efficiency of the fabricated biosensor in simultaneously identifying the mismatched or mutated OMP-M from non-mismatched ones (OMP-C).

In addition, the complementary characterization of surface modification of the Au electrode was illustrated by EIS (Fig. 4B). In the spectra of EIS at low frequencies, the Rct (charge transfer resistance) value as information of the diffusion [Fe (CN)_6_]^3−/4−^ redox couple toward the electrode surface is theoretically determined by a semi-circle diameter of the Nyquist plots. However, the Rct can be merely influenced by the establishment of a new layer on the electrode surface. As expected in the EIS curves, the straight line with a Rct value of only 22 Ω (curve a) was obtained for bare Au, confirming the existence of a quick charge transfer process of [Fe (CN)_6_]^3−/4−^ toward the electrode surface. When the OMP-SH probe was self-assembled onto the electrode surface followed by blocking with MCH, the Rct value was abruptly enhanced to 2428 Ω (curve b) and 3623 Ω (curve c), respectively. Evidently, it can be assumed that the SAM layer formation by the OMP-SH probe and the occupation of non-specific sites of the electrode surface by MCH make an electrostatic repulsive force to the electron transfer of [Fe (CN)_6_]^3−/4−^. As well, the more negative charges were induced by the hybridization of the probe with the target DNAs (1 µM) including OMP-M (with Rct value of 4885 Ω, curve e) and OMP-C (with Rct value of 5065 Ω, curve d), respectively. This supports the points to the fact that a double-strand DNA resulting from hybridization on the surface of OMP-SH probe is polymerized and thus, the Rct enhances owing to the pathway blockages for the redox probe availability toward the surface. The Rct of OMP-C hybridized was larger than that of OMP-M hybridized, associated with the fact that the number of formed hydrogens bound from base nucleotides of OM-C (20 bases) with OMP-SH probe is more than that of the OMP-M (17 bases) because of the having three-base mutants or mismatched in its sequence. In general, the results of EIS were in acceptable harmony with the data of SWV for electrochemical characterization at each step of modification at the electrode surface.

### Optimizing the experimental parameters

**Figure 5:**
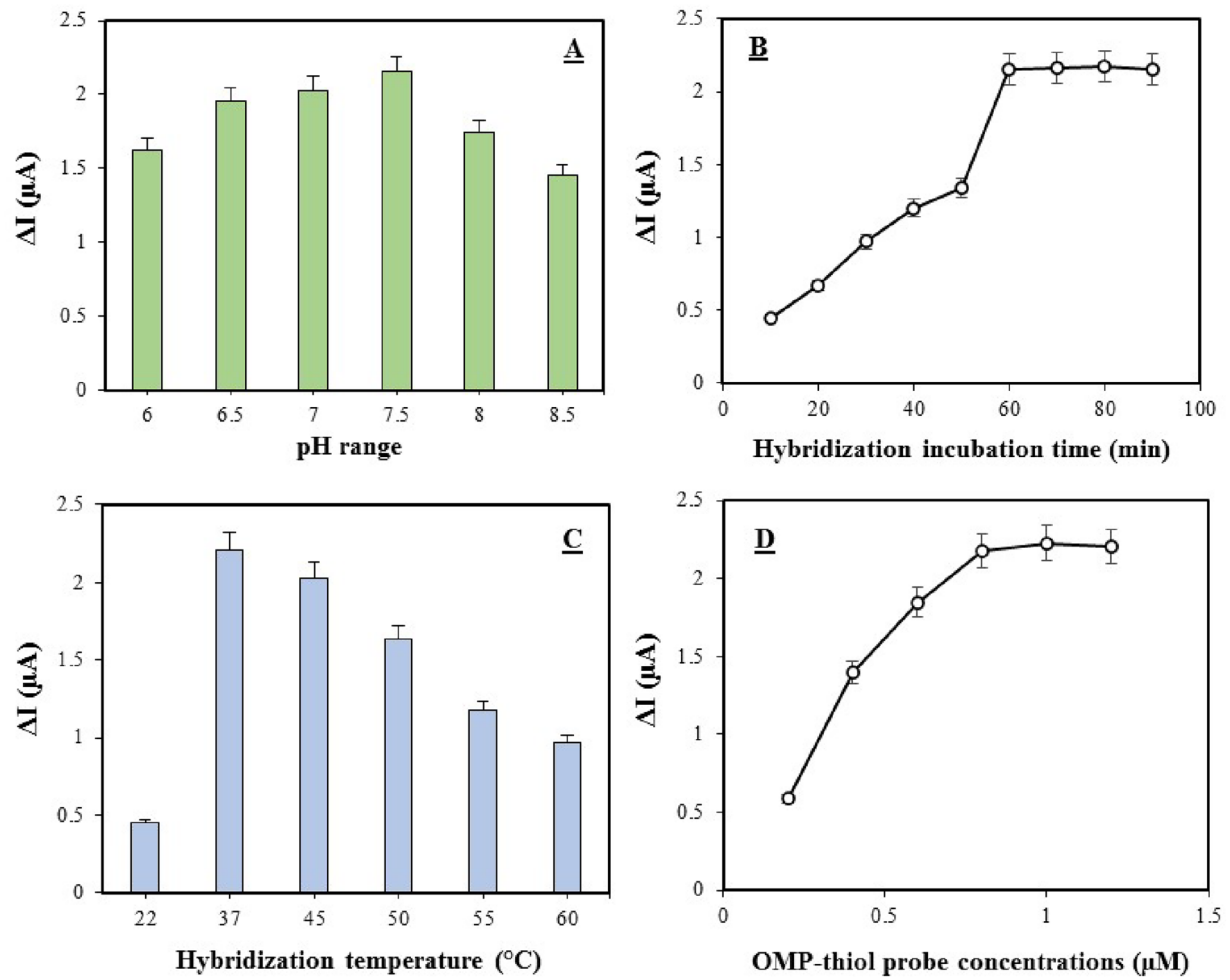
Optimization results of pH value (**A**), hybridization incubation time (**B**)**,** hybridization temperature (**C**) and the concentration of OMP-SH probe (**D**) as effective factors on performance of OMP-DNA probe biosensor.

To boost a to attain to maximum performance of the OMP-DNA probe biosensor, the optimization of the concentration of the OMP-SH probe (at 0.2–1.2 µM), hybridization time (at 10 to 90 min), hybridization temperature (at 22, 37, 45, 50, 55 and 60 °C) and pH (at ranging from 6 to 8.5) as effective factors for hybridization event were explored to determine. The dependence of the electrochemical response of the OMP-DNA probe biosensor (ΔI) at various pH incubations demonstrated that the current response promoted with increasing the pH value up to 7.5 and then declined at higher pH values (Fig. 5A). The efficient hybridization for the formation of duplex DNA complex was obtained at 7.5 pH, while the higher and lower pH value than 7.5 (alkaline conditions) caused the instability of hybridization. Hence, the pH value at 7.5 was chosen as the optimal condition for the formation of stable hybridization. For optimizing the incubation time and temperature of hybridization, when the incubation time and temperature of hybridization increased up to 60 min and 37 °C, respectively, ΔI displayed the highest current and optimal conditions for hybridization formation (Fig. 5B,C). But with further increase in temperature, ΔI declined, associated with instability of hybridization at higher temperatures that might affect the dynamics of hybridization. Also, with further increase in incubation time, the constant trend was observed for ΔI, which indicates that hybridization is completely formed and there is no any electrochemical response to the increase in incubation time.

**Figure 6:**
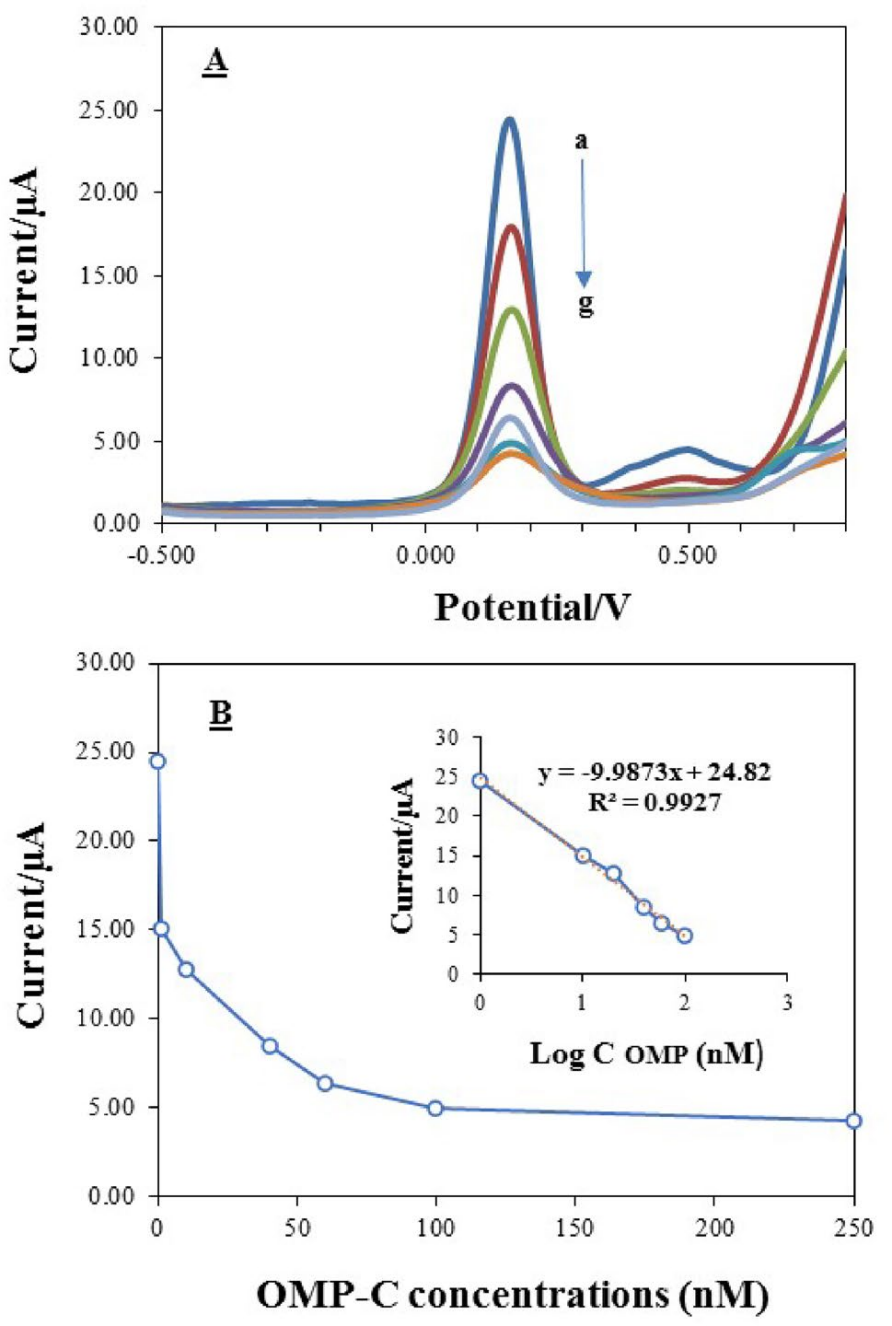
(**A**) Square wave voltammogram attained at OMP-SH probe/MCH/Au electrode after incubation with 0 nM (curve a), 1 nM (curve b), 20 nM (curve c), 40 nM (curve d), 60 nM (curve e), 100 nM (curve f), 250 nM (curve g) of OMP-Complement oligonucleotide in PBS solution, pH 7.5, containing 250 mM KCl and 5mM K_3_[Fe (CN)_6_] and 5mM K_4_[Fe (CN)_6_]. (**B**) Electrochemical OMP-DNA probe biosensor response as function of varying OMP-Complement concentration in the range of 1–250 nM. The insert indicates linear relationship among the response of OMP-DNA probe biosensor (µA) and OMP-complement concentration (nM) in the same range.

Moreover, SWV analysis was conducted by incubating the different concentrations of the OMP-SH probe (0.2–1.2 µM) onto the Au electrode in the electrode buffer for the formation of the SAM layer. Figure 5D demonstrates the resulting voltammogram. It was observed that the ΔI value indicated an increase with in increasing OMP-SH probe concentration in incubation solution, however, the maximum of ΔI was reached up to 1 µM concentration of probe and then displayed a constant trend. This indicates that 1 µM concentration of probe (ssDNA thiol) can react optimality with Au electrode surface for forming a stable SAM layer and a successful immobilization. In general, the pH value at 7.5, hybridization incubation time at 60 min, hybridization temperature at 37 °C, and 1 µM concentration of OMP-SH probe were chosen as optimal conditions in the following experiment for the performance of the fabricated biosensor. Accordingly, the 100 nM concentration of OMP-C **was** introduced as the optimal concentration for the high performance of the OMP-DNA biosensor, as shown in Fig. 6.

### Analytical performance of purposed biosensor

#### Sensitivity of the OMP-DNA biosensor

For the sensitivity investigation, the OMP-C concentrations (0–250 nM) were cast onto working electrode, and the electrochemical was response recorded by SWV, as shown in Fig. 6A. The resulting SWV voltammogram revealed that along with the increase of OMP-C concentrations, the height of peak currents decreased (Fig. 6A, a–g curves). It is obvious that the rate of hybridization formation and restructure of the duplex DNA molecule is promoted at the electrode surface with increasing OMP-C concentrations. Therefore, the results of the calibration plot found a logarithmic relationship between electrochemical response and OMP-C concentrations in the range of 1 to 100 nM, with a correlation coefficient of 0.992 and % RSD values lower than 2.28% (n = 3) (Fig. 6B, the inset of Fig. 6B). Additionally, the limits of detection (LOD) and quantification (LOQ) were predicted by using the equations: LOD = 3 s/S and LOQ = 10 s/S, where S and s represent the slope of calibration curve and the standard deviation of the response, respectively^37^. The result was predicted to be 0.026 and 0.089 nM for the LOD and LOQ values according to three times S/N ratio (signal-to-noise), respectively. These findings indicate the high sensitivity of the biosensor in forming hybridization and identifying the complementary strand DNA at the lowest concentration via the good orientation and the large specific surface area of OMP-SH/MCH/Au electrode for OMP-C attachment.

#### Selectivity, reproducibility, and stability of the OMP-DNA biosensor

**Figure 7:**
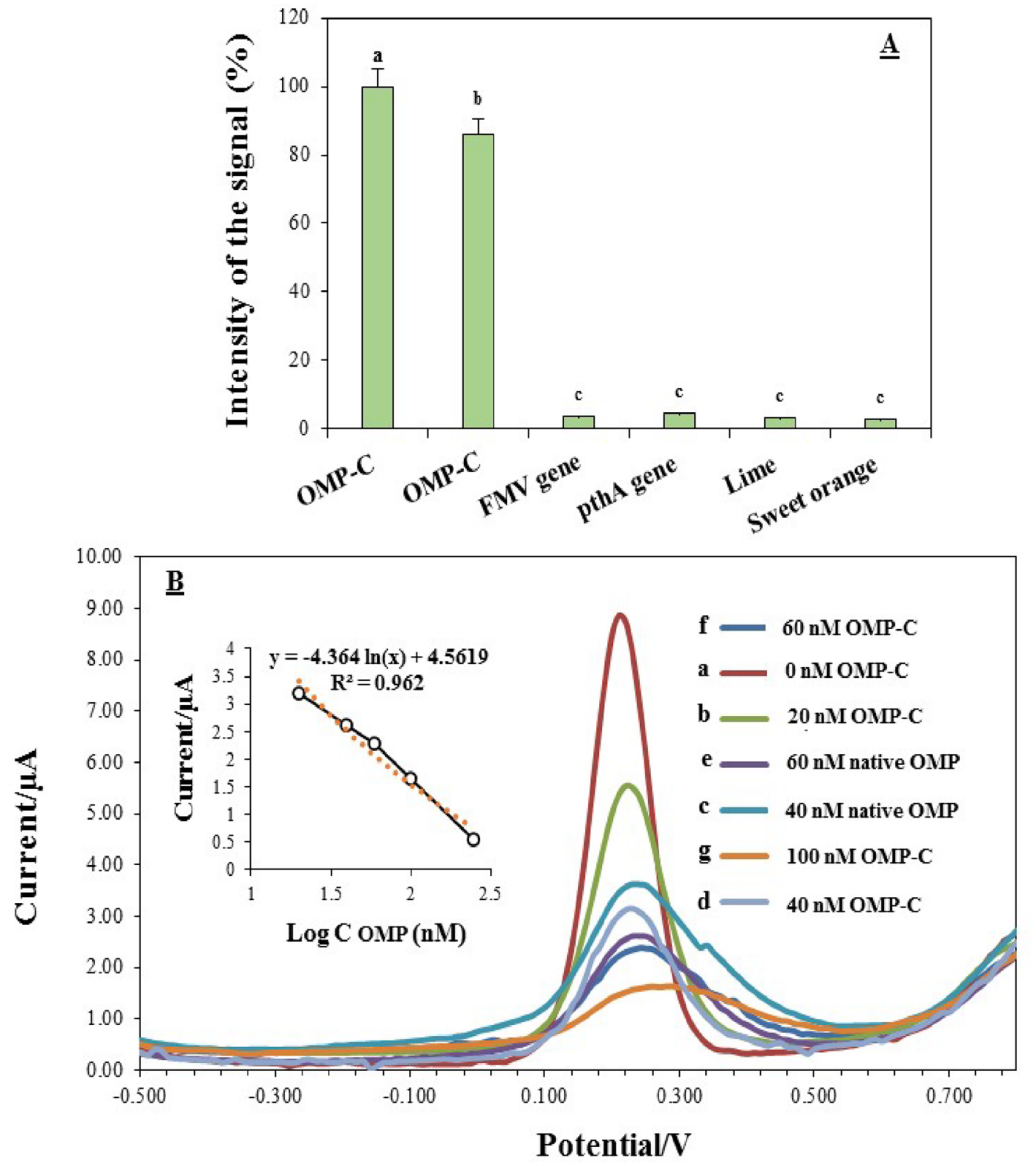
(**A**) The relative magnitudes of signals achieved from the OMP-DNA probe biosensor responses after incubation with 100 nM of OMP-C, 20 nM of NC gene (nucleocapsid protein gene of tomato yellow ring virus), 20 nM PthA gene, 20 nM fig mosaic virus (FMV) gene and 1 mM of DNA genomic extracted from healthy lime and sweet orange plants. (**B**) Square wave voltammograms of OMP-SH/MCH/Au electrode after incubation with (a–g curves): 40 nM and 60 nM of real samples (The native OMP-DNA extracted from the HLB-infected orange plants), 0 nM (OMP-DNA artificial sample provided from healthy plant without OMP-complementary), 20 nM, 40 nM, 60 nM and 100 nM of OMP-complementary spiked in healthy DNA from citrus plant samples. The insert indicates a linear relationship between the response of OMP-DNA probe biosensor (µA) and OMP-complement concentrations (nM) from these samples.

Furthermore, under the optimized condition, the selectivity of OMP-DNA probe biosensor for OMP-C identification was conducted **in** the presence of possible interferences from real samples or higher concentrations of other target DNAs including nucleocapsid protein (NC) gene of tomato yellow ring virus, fig mosaic virus (FMV) gene, PthA gene (as an effector protein of bacterial causal agent of citrus canker disease) and genomic DNA extracted from healthy lime and sweet orange plants. The resulting electrochemical response of OMP-C at a concentration of 100 nM was considered 100%; therefore, the signals obtained for interfering samples were compared with it. The results of variance analysis showed that no significant (at *p* < 0.01) responses were recorded for interfering samples, as shown in Fig. 7A. The electrochemical responses of less than 5% were detected for interfering samples. However, the largest or significant response belonged to OMP-C, which indicates that the fabricated OMP-DNA probe biosensor was adequately selective.

The reproducibility and stability were the next two factors to be examined. The biosensor reproducibility was conducted using 5 similarly prepared biosensors for the SWV analysis of 20 nM OMP-C. The RSD result, which was calculated to be 4.3% could be a sign of acceptable precision and reproducibility of the proposed OMP-DNA probe biosensor. Under the same experimental conditions, to investigate the long-time stability of five biosensors was also evaluated over 30-day period of time. The electrodes were kept at 4 °C in the absence of any other chemical preservatives during the measurement and their SWV signals were recorded towards OMP-C 20 nM every 10 days. It was revealed that OMP-DNA probe biosensor response sustained percentages of 96%, 93%, and 89% of the original value, respectively. The attained results confirmed the efficient storage stability of the OMP-DNA probe biosensor. Overall, the selectivity, reproducibility, and stability of the OMP-DNA probe biosensor based on an OMP-SH probe/MCH/Au are all reasonable, hence, it has the capability for quantitative detection of native OMP protein in real samples from HLB-infected citrus plants.

#### Real sample analysis: detection of native OMP gene in infected-HLB plant

The presence of the native OMP gene (878 bp) in citrus samples infected by HLB was successfully confirmed by PCR amplification using specific primers (data not shown) and thus, these confirmed-genomic DNA were subjected to the proposed biosensor. For exploring the applicability of the OMP-DNA probe biosensor to OMP detection in real samples, the real DNA sample containing native OMP at 40 nM and 60 nM (the OMP concentrations were measured by NanoDrop 2000/2000c UV–Vis Spectrophotometer after DNA extraction from HLB-infected citrus plant) following by the artificial infected sample, by spiking different concentrations of OMP-C (0 nM, 20 nM, 40 nM, 60 nM, 100 nM,) into DNA sample extracted from healthy sweet orange plant, were casted onto OMP-SH/MCH/Au electrode. The outcome signals of artificial samples disclosed a linear relationship among normalized current peak (µA) and log (C _OMP-C_) (nM), as can be observed in the inset of Fig. 7B. Comparative analysis detected that the slope of mentioned-linear relationship (4.46) from artificial samples was lower than the slope attained from OMP-C samples (9.98) in PBS buffer, which means the efficiency of hybridization formation and thus, the sensitivity of OMP-DNA probe biosensor in real sample can be influenced by the biological matrix. Even, this biological matrix effect led to an increase in the peak flow of bare gold from 2.5 (in PBS buffer) to 8.89 (in artificial sample, curve a). Despite the presence of matrix effects, the SWV voltammogram indicated that no significant cross-reactivity between the biosensor and possible interferences from DNA extracted from the sap plant (Fig. 7B). Hence, the peak of the distinct current was obtained for two HLB-infected samples at 40 nM and 60 nM concentrations (curves c and e) compared to artificial samples of the same concentration (curves d and f). Although the increase in the current peak of the HLB-infected samples due to the matrix’s effects slightly predisposed effect on the sensitivity of the proposed biosensor, but it did not lead to false positive or negative responses. Overall, the OMP-DNA probe biosensor was able to detect the native and synthesized OMP oligonucleotide in HLB-infected and artificial samples in the presence of background signals resulting from the biological matrix.

## Discussion

It is well-documented that DNA sequence detection has a great potential for rapid or reliable diagnosis of plant pathogens even before any symptoms of a disease appear^38^. The DNA probes can be utilized for the early detection of various pathogens without the need to isolate them via microbiological culture in special media^39^. Fast and early diagnosis is very crucial for bacterial causal agents of HLB disease, which it is non-culturable in free media so that the greatest strategies can be conducted for disease management before disease symptoms. Newly, the electrochemical DNA biosensor is announced as a novel exploring DNA biosensing technology for rapid, sensitive, simple, and early detection of pathogens^40^. However, a rapid, high sensitivity, and reliable detection system based on the electrochemical OMP-DNA probe biosensor offered in the current work for the diagnosis CLas bacteria, one of the most perilous species causal agents of HLB disease.

The primary result of the CV voltammogram for characterizing of OMP-SH/MCH/Au electrode displayed a well-defined characteristic reversable peak for redox couple [Fe (CN)_6_]^3−/4−^ at the bare Au electrode. So, fluctuations in the electron transfer kinetics of this redox couple are considered as a principal index to evaluate the electrochemical behavior of electrode surfaces by cyclic voltammetry after each step of surface modification. The application of the mentioned redox couple to measure the alternation in the electrochemical behavior of modified electrodes has previously been reported in the literature^20,41^. In contrast, the modification of the Au surface by MCH and OMP-SH resulted in less faradic currents and more peak separation (ΔEp) among the cathodic and anodic waves compared to the bare electrode. This is an attired-stable form of the SAM layer of OMP-SH-MCH at the surface of a clean Au electrode. It has been proved that the thiol group can promote the stability of monolayers by acting as a link for metallic attachments^36^. MCH, with both thiol and carboxylic acid groups, improves the immobilization stability in the following two ways: 1) the covalent reaction of its carboxyl group with the phosphate backbone of DNA and 2) acting as a spacer thiol that avoids the ssDNA from adhering to Au via their nitrogenous bases^36,42^. The covalent immobilization of DNA probe among thiol-modified DNA probes and Au surface for the SAM formation to fabricate biosensor has been applied in other studies^40,42,43^. Also, SWV analysis was conducted as a complementary technique in order to confirm the cyclic voltammetric analysis data, which showed a decrease or smaller peak current for the OMP-SH-MCH layer. The explanation for this smaller peak current could be associated with occupying the vacancy of the electrode surface by MCH and along with the formation of OMP-SH-MCH layer, it can reduce the electron transfer of redox couple [Fe (CN)_6_]^3−/4–^ toward the electrode surface through the creation of an electrostatic repulsion force between redox couple and the negatively charged phosphate backbone of DNA to Au^43^. Moreover, the peak currents were further diminished after DNA-DNA hybridization between the OMP-SH probe and target OMP-C due to the introduction of more negative charges by the hybridized target DNA. Ultimately, in this study, the redox peak current decrease is a good sign of the worth of the fabrication processes and certifies that all the ssDNA is anchored onto the Au surface only by the thiol end. Similar findings have been reported for the detection of *Bacillus* anthracis^36^, cancer diagnosis,^40^ and integral DNA recognition^43^ by hybridization event. Likewise, the EIS analysis, as a complementary technique, was conducted to confirm SWV results. When the surface modified by OMP-SH/Au, OMP-SH/MCH/Au, OMP-SH/MCH/OMP-M/Au and OMP-SH/MCH/OMP-C/Au electrodes, the diameter of the semicircle improved as well as Rct alternates to 2828 Ω, 3623 Ω, 4885 Ω and 5065 Ω, respectively, which supports the obtained results of SWV technique. This clearly shows that increasing the Rct value led to a limit in the diffusion of redox couple [Fe (CN)_6_]^3−/4−^ at each step of modification. Overall, it can be concluded that the simultaneous distinguish between OMP-C and OMP-M and the sensitivity of the fabricated biosensor in SWV is higher than that in the EIS technique. Accordingly, SWV was conducted to optimize the experimental parameters.

To attain to the high performance of fabricated biosensor in the detection of target DNA by hybridization mechanism, the optimization of some critical experimental factors including the immobilization concentration of the OMP-SH probe, pH, reaction time**,** and hybridization temperature was investigated. Owing to the nature of the measurement procedure, the sensitivity of the proposed biosensor might be influenced by the pH of the sample solution which can affect the amount of hybridization of the target DNA (OMP-C ssDNA) with the surface of OMP-SH/MCH/Au electrode. Hybridization efficiency in surface-based biosensor is more dependent on the accessibility and binding affinity of DNA and thus, the better efficiency of hybridization will be associated with proper accessibility^44^. The pH value of 7.5 indicated the highest current peak (ΔI) which resulted in the most hybridization of target OMP-C. The lower signal flow at a lower pH value and higher than 7.5 may be attributed to reduce the accessibility of bound ssDNA probe for hybridization by tensile surface stress due to hydration and electrostatic induced forces^44,45^. So, the effect of probe concentrations was investigated by using the SWV technique in a solution buffer at pH = 7.5. Immobilization conditions like interfacial electrostatic potential and ionic strength affect the probe density, which also affects the efficiency of hybridization^39^. The concentration of 1 µM OMP-SH probe displayed the largest ΔI which enhanced the assembly amount of probe DNA to make more amounts of DNA hybrids on the electrode for a larger electrochemical response. In contrast, no increase in signal flow was electrochemically detected when the OMP-SH probe at 10 µM or higher density cast onto a working electrode (data not shown). It can be elucidated by the lower accessibility of the target DNA and massive probe accumulation on the surface of the electrode that induces probe overloading. This case can be resolved by blocking the MCH on the surface electrode. Actually, MCH can prevent the formation of tightly packed probe DNA monolayer via dropping steric and electrostatic hindrances and can also provide a better position hybridization by regulating ssDNA-thiol probe anchoring^36,46^. These results are in agreement with the findings of Zhang et al.^42^, Chen et al.^43^, and Mahmoodi et al.^40^. According to the Langmuir-like kinetic, a low probe density permits 100% of the probe to hybridize while a high probe density can drop to 10% of the probe hybridization^47^. As a result, the concentration of 1 µM OMP-SH probe was chosen as the optimal concentration for further electrochemical analysis to determine the optimal time and temperature of hybridization. The hybridization time of 60 min showed the largest ΔI and afterward, no change in the current flow was observed. Also, the high hybridization and electrochemical signals happened to reaction temperature at 37°C, and afterward, the electrochemical responses were gradually decanted at higher reaction temperatures. It has been distinguished that low temperatures of hybridization drive the formation and enhance stability of large aggregating species while high temperatures destabilize base-pair interactions and large aggregates. However, the optimal range of moderate temperatures permits the minimization of large aggregate formation and maximization of fully hybridized dimers^48^.

Analytical performances of the OMP-DNA probe biosensor were determined by specificity, sensitivity**,** and selectivity of hybridization with target DNA under the optimal experimental conditions via the SWV technique. The specificity and selectivity of the fabricated biosensor was distinguished by hybridizing the OMP-SH/MCH/Au electrode with the various target DNAs. During hybridization, the obtained signal from electrochemical behavior was gradually decreased for the immobilized OMP-SH probe, OMP-M**,** and OMP-C oligonucleotides, respectively. A lightly higher current peak than OMP-C was recorded for OMP-M containing three mismatch DNA, which might be described as follows: the immobilized OMP-SH probe failed to form the complete hybridization with OMP-M. This result is in agreement with findings of Mahmoodi et al.^40^. This means that the designed OMP-DNA probe biosensor has high specificity in distinguishing the three-base mutation oligonucleotides or random oligonucleotides from complementary target DNAs. However, it can be utilized for selective detection of target DNA from among multiplexed DNA sequences of non-target species. The result of the electrochemical responses in the presence of an interference sample revealed that no overlap is significantly detected among the target OPM-C and any potential cross-reaction with other DNA sequences (*p* < 0.01).

The detection sensitivity of the fabricated OMP-DNA probe biosensor toward the concentrations of target OMP-C at ranges of 0–250 nM was examined. The calibration plot calculated the LOD and LOQ by 0.026 and 0.086 nM, respectively, which such detection limit was greater or comparable with other testified PCR-based techniques for HLB detection^26^. The RSD for the detection of 1 nM, 10 nM, 20 nM, 40 nM, 60 nM**,** and 100 nM were predicted as 4.23, 3.38, 2.18, 2.15**,** and 2.38% based on three replicated electrochemical measurements. This confirms that the fabricated DNA biosensor has an acceptable reusability and reproducibility for detection. Hence, to attain the applicative potential of the proposed DNA biosensor in the presence of relatively complex biological samples, detections of OMP-C spiked in DNA extracted from a healthy citrus plant (artificial sample) were examined. Although the artificial sample induced some effect on the background and the signal response, the electrochemical responses were still dependent on the spiked OMP-C concentration in healthy genomic DNA. The background response and signal response in artificial sample were larger and mostly lower than in the solution buffer, respectively. The explanation for the obtained background response may be attributed to the non-specific adsorption of some biological molecules in the artificial sample on the electrode. Chen et al.^43^ also attained parallel findings for background response in the fabrication of DNA biosensor, supporting the current study. To estimate the general applicability of the OMP-DNA probe biosensor in real sample analysis, the genomic DNA extracted from HLB-infected citrus plant containing the native OMP gene at 40 and 60 nM concentrations was cast on the working electrode. The signal response and background response in the real sample were slightly larger than artificial sample at the same concentrations due to the biological matrix. However, the signal response was DNA concentrations and there were no observed false positive and negative responses. Increasing current peak in real samples due to biological matrix has previously been stated for detecting other target molecules in other studies^20,22,23,43,49^. These outcomes proposed an applicative potential of OMP-DNA probe biosensor in relatively complex biological matrices.

Moreover, due to the importance of HLB detection in today’s research world, some relevant recent detection systems were considered to be compared with the introduced OMP-DNA probe biosensor (Table 1). Overall, various diagnosis systems such as serological-based antibodies, molecular-based PCRs, and biosensor-based HLB disease detection have been established for HLB detection^27^. To conserve HLB-free citrus-producing areas from attacking HLB bacteria, considering an early detection system is a fundamental implement to prevent the spread of HLB disease. For this goal, the advantages and disadvantages of recent diagnosis systems must be identified to boost the HLB management programs. Although molecular-based PCR methods are permitted to detect HLB pathogens at low concentrations, they are time-consuming, and expensive and can be applied to HLB detection at later stages of infection. In contrast, serological-based antibodies such as ELISA and dot blot are low-cost, rapid, and easy to use under field conditions, but they have low sensitivity for plant bacteria^12,27^. The gas biosensor arrays and microsensor based on detecting the volatile organic compound biomarkers and limonin biomolecule are more sensitive to HLB detection compared to serological assays, but they have some limitations that led to a decrease in their analytical performance to detect target pathogens. The quick stimulation of citrus host plant’s volatile compounds against abiotic and biotic stresses, lack of synthesis of the biochemical biomarkers in most citrus varieties, and differing biochemical contents released by HLB-infected citrus due to various responses of different citrus varieties against HLB stress under environmental condition are most limitations of mentioned biosensors to HLB detection in indirect way^27,50^, which decline their sensitivity, specificity**,** and popularity to use to HLB detection. Chemiresistive biosensor based on secreted proteins of CLas is more sensitive and specific than the mentioned biosensors-based biochemical biomarkers, which enable to detection of the HLB pathogen in a direct way, while its sensitivity **is** remarkably lower than PCR methods. Despite the high sensitivity of chemiresistive biosensor, its analytical performance has strangely declined due to various factors including different expression levels of the secreted proteins in the host citrus and the vector psyllid, secreted protein fluctuations in different plant organs due to HLB bacterial migration under seasonal and temperature changes, the presence of a very low level of secreted proteins in the phloem tissue due to the lack of HLB bacterial activity under inappropriate temperature and seasonal conditions and the possibility of not identifying the secreted proteins by the antibody of the biosensor system due to the deformation structure of target protein form in the pathways of the resistance mechanism of the host plant. Based on the chosen literature records, it could be noticed that the OMP-DNA probe biosensor has presented such **a** low detection limit besides a wide linear range of response, while its fabrication does not include nor complex neither time-consuming procedure and its simplicity could be considered as the most important advantage of it.

**Table 1:** Comparison between the present study and some recent applied immunoassays for HLB disease detection.

Immunoassay type	Detection system	Measured analyte	Linear range	LOD	Refs.
ELISA-based on polyclonal antibody	Direct way	Native and recombinant OMP protein	1–800 µg/µl	6 µM	^16^
ELISA-based on scFv antibody	Direct way	Genes encoding proteins on CLas surface	0–50 µg/µl	0.50 µM	^17^
Conventional PCR	Direct way	DNA genomic of CLas	1–20 ng/µl	1 nM	^10^
qPCR	Direct way	DNA genomic of CLas	1–20 ng/µl	0.10 nM	^10^
Nested PCR	Direct way	DNA genomic of CLas	1**–**20 ng/µl	0.010 nM	^10^
Micro sens or	Indirect way	Limonin biomolecule	NA	10 nM	^26^
Gas biosensor arrays	Indirect way	Volatile organic compound biomarkers	NA	NA	^25^
Chemiresistive biosensor	Direct way	Secreted protein biomarker	NA	5 nM	^24^
The purposed biosensor	Direct way	OMP-DNA probe	1–250 ng/µl	0.026 nM	This work

*NA* Non-available, *scFv* single chain variable fragment (scFv) antibodies.

## Conclusion

The current study offered the simple and sensitive electrochemical DNA biosensor for early detection of CL. asiaticus as the causal agent of citrus greening disease. The SEM image, CV, EIS**,** and SWV methods were applied to morphological and electrochemical characterization of the individual steps of the OMP-DNA probe biosensor fabrication. According to outcomes from electrochemical response during hybridization, it can be concluded that: (1) The proposed biosensor indicated a high specificity in base mutation analysis and also, a good selectivity without the obvious cross-interference for target DNA detection among million-fold excess of non-target sequences by hybridization, which confirms the high performance of the biosensor. (2) the sensitivity of biosensor displayed a much lower detection limit (0.026 nM) compared with other reported PCR-based techniques and immunoassay methods. (3) It also has high applicative potential for DNA detection in a relatively complex biological matrix (healthy genomic DNA) and real sample (OMP-DNA extracted from HLB-infected citrus plant). Therefore, the developed label-free electrochemical OMP-DNA probe biosensor can be utilized as an effective tool for rapid, accurate, easy, and early detection of CL*. asiaticus* for screening citrus greening under field conditions in the near future.

## Methods

### Chemical material and instrumentation

Bovine serum albumin (BSA), potassium ferricyanide (K_3_Fe (CN)_6_), potassium ferrocyanide (K_4_Fe (CN)_6_), potassium chloride (KCl), 6-Mercapto-l-hexanol (MCH) and Tween-20 were supplied from Sigma-Aldrich chemicals, and used without any further purification. Phosphate buffer saline solution (PBS) (consisted 10 mM Na_2_HPO_4_, 10 mM KH_2_PO_4_ and 250 mM NaCl, pH 7.4), and PBST (consisted PBS solution and 0.05% W/V Tween 20, pH 7.4) were provided using distilled water as electrode buffer and washing solution, respectively. PalmSens potentiostat (Palm Instrument BV, the Netherlands) was applied to measure the voltammetric experiments including cyclic voltammetry (CV), square wave voltammetric (SWV), and electrochemical impedance spectroscopy (EIS) as electrochemical measurements at room temperature. All measurements were conducted at a conventional three-electrode system consisting of a gold disk electrode (Au with 3 mm diameter, CH Instruments Inc., Austin, TX as working electrode), Ag/AgCl (as reference electrode), and a platinum wire (Pt as counter electrode).

### OMP-DNA probe and target sequence selection

In this study, a DNA probe was designed for the OMP (outer membrane protein; accession No. AY842432) gene from *Ca. L. asiaticus* genome, which was obtained from the National Center for Biotechnology Information (NCBI) Gene bank (www.ncbi.nlm.nih.gov). All DNA oligonucleotides were synthesized by MetaBIOn International AG Company (Bayern, Germany) as lyophilized powder. The oligonucleotide sequences were listed as follows:

1. OMP-SH probe (thiolated OMP-probe sequence): 5′-SH-(CH_2_)_6_ -GCAGCCGCAGATCGAGAGCG-3′
2. OMP-C (OMP complement): 5′- CGCTCTCGATCTGCGGCTGC-3′
3. OMP-M (OMP mutation consisted of three-base mismatched DNA or three points mutation of OMP sequence): 5′- CGCAGTCGATCTGCGGCAGC-3′

The underline shows the mismatched bases of the OMP-C sequence. The oligonucleotide stock solutions and more dilute solutions of targets (100 µM) were prepared with TE buffer (1 M Tris-HCl (pH 10) and 0.5 M EDTA with pH 8.00) and kept frozen.

### Working electrode preparation

Gold electrodes were cleaned by physical and chemical techniques following a described protocol^20,22,23^. To attain a clean Au electrode surface, it was mechanically polished with 0.3 and 0.05 μmalumina slurry, respectively, then washed between each polishing step with ultrapure water for 2 min. Then Au was electrochemically cleaned by incubating in piranha’s solution (1:3, 30% H_2_O_2_ and concentrated H_2_SO_4_, respectively) for 5 min at 60 °C then rinsed with deionized water. The chemical polish was repeated twice. Finally, the electrode was more electrochemically cleaned by placing it in a freshly prepared 0.5 M H_2_SO_4_ solution before DNA probe immobilization and cycled in the potential range of 0.0 to 1.5 V versus Ag/AgCl reference electrode with a scan rate of 0.1 Vs^−1^ for 25 scans. In the end, the Au electrode was rinsed with deionized water and dried with nitrogen gas.

### Fabrication of the electrochemical OMP-DNA biosensor

A schematic diagram representing the main steps in the fabrication of the electrochemical OMP-DNA probe biosensor in this study is indicated in Fig. 1. For the reproducible formation of SAM layers, at first, 2 µl (1 µM) OMP-SH probe was only dropped on cleaned Au electrode surface and then was incubated in the moist environment of culture disk in the water bath at 37 °C for 6 h to self-assemble and formation a stable Au–S bound. After the chemisorption attachment of the DNA probe via S–H to the metal surface of the Au electrode, the unbounded molecules or weakly adsorbed OMP-SH probe was removed by rinsing with washing solution from the Au surface. Secondly, the modified electrode (OMP-SH/Au) was incubated by 25 µl of 1.0 mM MCH for 30 min to block the un-assembled surfaces. Lastly, the immobilization of the OMP-SH probe was perfected by rinsing the Au electrode with a washing solution thoroughly and was stored in the refrigerator when not in use. Electrodeposition of targes DNA oligonucleotides and morphological characterization of the modified Au electrode surface was carried out by CV (in the potential range of − 0.5 to 0.7 Vs^−1^ with a scan rate of 0.05 Vs^−1^ for 25 scans) and EIS (with a frequency range of 1 Hz to 1 MHz at DC potential of 0.2 V with AC amplitude of 0.01 V) measurements.

### Hybridization and electrochemical detection of target DNA

The DNA biosensor fabricated (OMP-SH/Au) with the above-mentioned protocol was designed to form a free-label electrochemical OMP-DNA probe biosensor based on the changes of electrochemical behavior with or without the presence of complementary DNA target. The electrochemical signal was detected based on the electroactive DNA bases that underwent a reduction and oxidation reaction after hybridization. 20 µl of a fixed concentration (100 nM) of target DNAs solution including OMP-C and OMP-M was dropped on the surface of the OMP-SH/Au modified electrode, and allowed to hybridize in a water bath at 37 °C for 60 min prior to rinsing the electrode with PBST washing solution. After hybridization, the resulting modified electrode (OMP-C/OMP-M/OMP-SH/Au) was subjected to electrochemical measurements in the next step. The SWV measurement was conducted in the potential range from − 0.5 to − 0.1 V with an amplitude of 0.05 V and a frequency of 15 Hz in the electrolyte buffer. The analytical outputs of hybridization efficiency of target ssDNAs with OMP-SH probe were considered by a decreased average of oxidation peak currents at OMP-SH/Au electrode surface ΔI (ΔI = I_0_ − I), where I_0_ and I was the oxidation peak current before and after hybridization, respectively. All electrochemical experiments were accomplished in 5 mM [Fe (CN)_6_]^3−/4−^ and 250 mM KCl and were performed at room temperature. After each measurement, the OMP-DNA probe biosensor was rinsed with PBST solution.

### Electrochemical screening of HLB-infected citrus plant

HLB-infected and healthy citrus plants (sweet orange) were collected from a citrus orchard located at Zakin, Minab city, Hormozgan province of Iran. The midribs and fruit peels were excised. Then, 100 mg of each sample was crushed in liquid nitrogen, and DNA was extracted via the CTAB (Cetyltrimethyl ammonium bromide) method as previously described^4^. The PCR reaction was carried out using OI1/OI2c and A2/J5 primers to molecular identify the citrus plants infected by CL. asiaticus according to the previously protocol described by Ruangwong and Akarapisan ^51^. The HLB-infected-samples confirmed by OI1/OI2c and A2/J5 primers were used for genomic DNA extraction to amplify of OMP gene (Data not shown). The PCR products were analyzed by gel electrophoresis using a 1% agarose and then visualized by the GEL documentation (SYNGENE; GENE Genious Bio Imaging System). The confirmed DNA genomic containing the native OMP gene was dropped on the OMP-SH/Au modified electrode surface to hybridize. The electrochemical response or signal of their hybridization was measured by the SWV experiment.

### Ethics approval

The authors declare that all experimental citrus plants used in this study were collected under a protocol approved by the Citrus and Subtropical Fruits Research Center of Iran and were conducted in accordance with its relevant guidelines and regulations. Also, the authors confirm that the citrus plants used in this study are not endangered species of wild citrus.

## Data Availability

The authors confirm that the datasets analyzed during the current study are available from the corresponding author upon reasonable request.
